# The Relationship between Food Insecurity and Symptoms of Attention-Deficit Hyperactivity Disorder in Children: A Summary of the Literature

**DOI:** 10.3390/nu11030659

**Published:** 2019-03-19

**Authors:** Stacy Lu, Leanna Perez, Abby Leslein, Irene Hatsu

**Affiliations:** 1 Department of Human Sciences, Ohio State University, Columbus, OH 43210, USA; lu.1245@buckeyemail.osu.edu (S.L.); perez.264@osu.edu (L.P.); 2 Department of Neuroscience, Ohio State University, Columbus, OH 43210, USA; leslein.13@buckeyemail.osu.edu; 3 OSU Extension, Ohio State University, Columbus, OH 43210, USA

**Keywords:** food insecurity, food insufficiency, hunger, attention-deficit hyperactivity disorder, ADHD

## Abstract

Food insecurity is a major public health concern characterized by an individual or household lacking access to adequate food to support a healthy lifestyle. Food insecurity has been associated with predisposing or exacerbating mental health symptoms in children. However, the evidence is scarce with regards to Attention-Deficit Hyperactivity Disorder (ADHD) symptoms in children. The purpose of this review is to summarize and identify gaps in the existing literature, as well as to explore associations between food insecurity and symptoms of childhood ADHD. Literature for this review was pulled from Ovid MEDLINE and PubMed library databases, with a focus on food insecurity, food insufficiency, hunger, and ADHD symptoms such as inattention, hyperactivity, and impulsivity in children. The limited evidence to date shows a predictive and inverse relationship between childhood experience of food insecurity and symptoms of ADHD, with lasting impacts into adulthood. Evidence exists to hypothesize that childhood food insecurity is associated with predisposing or exacerbating ADHD symptoms in children, yet the literature needed to confirm this relationship is scarce and utilizes inconsistent methodology. Future research is needed to further characterize this complex relationship and inspire community or public health interventions addressing food insecurity in children with ADHD. Additionally, it may be clinically useful to routinely screen for food insecurity when assessing pediatric ADHD symptoms.

## 1. Introduction

As of 2017, approximately 12.5 million children in the United States lived in food insecure households [1]. Food insecurity is characterized by an individual or household lacking access to adequate food to support a healthy lifestyle [1]. Food insufficiency, on the other hand, is characterized by an inadequate amount of food intake due to a lack of money or resources [2]. Hunger is another term frequently associated with a lack of food. It is defined as “short-term physical discomfort as a result of chronic food shortage, or in severe cases, a life-threatening lack of food” [3]. Although food insufficiency and hunger are often used interchangeably with food insecurity, the terms are not synonymous. Regardless, lacking consistent, safe, and sufficient food to support a healthy and active lifestyle is accompanied with stress on an individual or the household.

While there is evidence of an association between food insecurity and childhood mental health disorders, limited studies have focused on how food insecurity may affect disorders such as Attention-Deficit Hyperactivity Disorder (ADHD). ADHD is a neuropsychiatric disorder commonly diagnosed in childhood with increasing global prevalence and high heritability [4]. Symptoms of inattention, impulsivity, and hyperactivity are characteristics of ADHD, impairing an individual’s ability to function in multiple settings such as at home and at school or work [5]. The current prevalence of ADHD diagnoses in the US is 10.2%, with an estimated 70% to 80% heritability [4,6,7]. The increasing prevalence and high heritability of ADHD diagnoses among children makes this condition a public health concern.

Despite the high prevalence of childhood food insecurity [8] and its possible effects on mental health, as well as high prevalence of pediatric ADHD diagnoses [4,8], the literature exploring the interactions between these public health crises is limited. Evidence exists to show that early childhood exposure to environmental stressors, including food insecurity, could be associated with predisposing or exacerbating ADHD symptoms in children [9,10]. Several studies have found behavioral challenges to be associated with periods of compromised nutrition during critical periods of early childhood development [9,11,12,13]. In addition to immediate impacts on children’s externalizing behavior, there is growing literature indicating long-term behavioral effects of early childhood exposure to food insecurity [9,14,15,16,17]. For example, inattention and impulsivity may continue, and sometimes intensify, into adolescence and adulthood [9,14,15,16,17]. Thus, early interventions targeting childhood food insecurity may also affect the occurrence and severity of future behavioral problems. The purpose of this review is to summarize and identify gaps in the current literature exploring the relationships between food insecurity and early childhood ADHD symptom prevalence and severity.

## 2. Materials and Methods

A comprehensive literature search was conducted to identify all studies investigating the associations between food insecurity and symptoms of ADHD. Online databases including Ovid MEDLINE and PubMed were utilized to complete this review. In addition, a hand search was conducted by reading through citations of articles that were found. The key search terms used to identify relevant articles were: attention deficit hyperactivity disorder, ADHD, inattention, hyperactivity, impulsivity, mental health, hunger, food insecurity, food insufficiency, food neglect, children, and pediatric. Exclusion criteria included articles examining the associations between food insecurity and symptoms of inattention in children with intellectual disabilities or severe childhood malnourishment. The focus of these articles did not fit the scope of this review and there may be alternative relationships between severe malnutrition and inattention in children, such as an identified intellectual disability.

## 3. Results

Results using these terms yielded 23 articles. The review includes publications from 1998 to 2016. Articles examining intellectual disabilities (*n* = 3) or severe childhood malnourishment (*n* = 9) were excluded, in addition to duplicates. Following this exclusion criteria, 11 articles remained and were included in the review. All of these studies, except one, were conducted in the United States or Canada and included children with an age range of 0 to 17 years. In addition, 8 out of the 11 studies utilized fairly large sample sizes (*n* = 2120–34,427). Table 1 and Table 2 summarize the articles mentioned in the text, where they are grouped into respective categories based on whether studies focused on food insecurity or hunger.

### 3.1. Evidence to Date

#### 3.1.1. Food Insecurity and ADHD Symptoms

Table 1 presents the results from the literature search for food insecurity and ADHD symptoms in children. Several large-scale epidemiological, observational, and interventional studies have examined the relationship between the presence of childhood food insecurity and symptoms of ADHD, particularly with behavioral problems such as hyperactivity and impulsivity, into adolescence and adulthood [15,17]. Specific characteristics of food insecurity, such as unreliable access to food and inadequate amounts of food intake, are most commonly identified in these studies as being related to behavior symptoms characteristic of ADHD. Among three-year-old children living in the USA (*n* = 2870), a relationship between food insecurity and ADHD symptoms was found, where the severity of household food insecurity was significantly related to childhood behavioral problems such as hyperactivity and inattention [18]. Similarly, a study conducted in 2012 among American youth aged 3–17 years (*n* = 6483) experiencing food insecurity found a 14% odds increase of all past-year mental disorder diagnoses, including behavioral disorders such as ADHD [19]. Likewise, a 2016 study among American youth ages 12–16 years (*n* = 8600) identified food insecurity to be more strongly related to children’s mental disorders than traditional socioeconomic measurements, with food insecurity independently associated with over a two-fold increase in risk for mental health problems [21]. Specifically, parents rated food insecure adolescents higher for hyperactivity scores than adolescents who were food secure [21].

Several longitudinal studies have also examined the relationship between food insecurity and symptoms of ADHD. Among kindergarten-aged children in the USA (*n* = 6,300), the timing and duration of food insecurity was found to affect the display of behavior in children, with behavior problems such as self-control being more sensitive to transitions into food insecurity than chronic food insecurity [13]. A study of American children ages 4–14 years (*n* = 2,810) found persistent food insecurity and transitions into food insecurity predicted approximately two-times increased likelihood of exhibiting hyperactivity and/or non-compliance behaviors at follow-up [11]. Results from this study were not confounded by poverty and demonstrate the predictive power of food insecurity for children’s externalizing behaviors. Another study in Canada in 2012 analyzed the impact of food insecurity on ADHD symptoms among children ages 1.5–8 years (*n* = 2120). The study found food insecurity to distinctively predict a two-fold increased likelihood of persistent hyperactivity and inattention over the course of seven years among the children [12]. More importantly, this relationship remained elevated and significant after adjusting for child and family characteristics. However, the measures utilized in two of these studies were not validated to assess food insecurity and more accurately capture food insufficiency [11,12].

#### 3.1.2. Hunger and ADHD Symptoms

Key symptoms of ADHD include inattention, hyperactivity, and impulsivity, which are also behaviors often displayed by children who are acutely hungry. Table 2 presents the results from the literature search for hunger and ADHD symptoms in children. A study conducted in 2007 among Jamaican adolescents aged 17–18 years (*n* = 129) experiencing hunger found poorer psychological functioning in late adolescence, with parents reporting more hyperactivity [15]. Similarly, in a US sample of children ages 6–12 years (*n* = 328), hungry children scored significantly higher on psychosocial dysfunction and were three-times more likely to be classified as dysfunctional compared to non-hungry children [23]. More specifically, the hungry children displayed more inattentive, hyperactive, and impulsive behaviors [22]. Another study among American youth ages 6–12 years (*n* = 205), found hunger to negatively impact behavioral functioning based on parent reports on the Pediatric Symptom Checklist (PSC) and Child Behavior Checklist (CBCL) [24]. These are commonly used tools for pediatric psychiatric assessments including questions regarding inattention and hyperactivity. Teacher reports from this sample indicated higher levels of hyperactivity and attention problems in hungry children [23]. This relationship between hunger and symptoms of ADHD also seems to have lasting impacts into adulthood. A recent study in 2016 interviewing young adults ages 18 years and older (*n* = 34,427) found those who had experienced childhood hunger were two-times more likely to report a lifetime history of interpersonal violence and challenges with impulse control, indicating associations between frequent childhood hunger and later development of behavioral problems [24]. This study was also able to identify a direct path from frequent childhood hunger to later interpersonal violence, with impulse-control deficits as a mediating factor, while controlling for sociodemographic factors such as household income, race/ethnicity, and education [24].

## 4. Discussion

To date, several large-scale epidemiological, observational, and interventional studies support an inverse relationship between the presence of early childhood food insecurity and symptoms of ADHD [9,11,12,13,18,19,20]. The presence of food insecurity during early child development may also influence the persistence of mental health issues, particularly behavioral problems such as hyperactivity and impulsivity, into adolescence and adulthood [14,15,16,17]. Several pediatric studies have replicated correlational relationships between food insecurity and ADHD symptoms in the past decade. Specific characteristics of food insecurity, such as unreliable access to food and inadequate amounts of food, are most commonly identified in these studies as being related to behavior symptoms characteristic of ADHD. Previous research has also suggested that effects on behavior in youth become prevalent even at the marginal food insecurity level [20]. Additionally, there is some evidence indicating that the timing and duration of food insecurity can affect the presence of childhood behavior as they seem to be more sensitive during transitional periods into food insecurity rather than chronic periods of food insecurity [13]. Regardless of timing or duration, overall worse hyperactivity and mental health outcomes were reported in food insecure children [13].

Additional evidence has found that the relationship between food insecurity and symptoms of ADHD is a predictive, inverse relationship, even after adjusting for child and family characteristics [11,12,19,21,24]. However, determining a causality between food insecurity and externalizing behavior symptoms presents its challenges, especially given the additional environmental, household dynamics, and genetic associations implicated with early childhood development [10,25]. Nonetheless, some studies have reported strong associations between food insecurity and symptoms of ADHD while controlling for poverty, household income, parental education, parental mental health, and child’s descriptive characteristics [11,12,19,21,24].

The literature to date also suggests that food insecurity may also affect the development of mood dysregulation and problems with interpersonal skills in children displaying ADHD symptoms. Co-occurring mood dysregulation symptoms of irritability, anger, and aggression are often seen in children with ADHD [7]. Walker et al. reported hungry adolescents with greater tendencies for conduct disorder at age 11 and oppositional behavior at age 17 [14]. In another study by Poole-Di Salvo et al., food insecurity was independently associated with an increased risk for conduct problems and sub-optimal pro-social skills in adolescents [21]. Conduct problems in this study were characterized by externalizing behaviors including anger, aggression, and irritability while pro-social skills were characterized by behaviors that benefit others such as helping, sharing, and cooperating [21]. Similarly, Kimbro et al. reported poorer scores for interpersonal skills in children transitioning into food insecurity by teacher report [13]. Although impaired social function is not a key symptom of ADHD, symptoms such as inattention and impulsivity may negatively affect a child’s ability to communicate or interact with others [4]. These findings support previous findings that describe co-occurring mood dysregulation and social dysfunction commonly found in children with comorbid ADHD symptoms.

### 4.1. The Case for Consistent Methodology

#### 4.1.1. Food Insecurity Methodology

Studies included in this literature review utilized various measures of food insecurity, making it challenging to consistently and accurately identify individuals who live in food insecure households. Currently, the US Household Food Security Survey Module (USHFSSM) developed by the United States Department of Agriculture (USDA) is an industry “standard” for measuring the prevalence and severity of food insecurity in the United States and Canada [26,27,28,29]. As shown in Table 1, five studies in our review were conducted in the United States and Canada and utilized the USHFSSM to ascertain food security status [13,18,19,20,21]. The remaining two studies used methods such as single-question parent assessments and investigator-defined interviews [11,12]. The latter measures often assess food insufficiency instead of food insecurity, as they do not capture the four main types of situations associated with the general definition of food insecurity (i.e. anxiety about inadequate food supply, perceptions of inadequacy with food quality, and reported instances/consequences with reduced food intake among adults and children). A brief summary of these methods follows.

The USHFSSM is a questionnaire that assesses household food environment and challenges in meeting food needs over 12 months [28]. This questionnaire is validated in both the United States [26,27,28] and Canada [29] to measure household food insecurity. Respondents are categorized into “High Food Security”, “Marginal Food Security”, “Low Food Security”, and “Very Low Food Security” based on the summation of raw scores. The categorization of score ranges varies depending on whether or not there are children present in the household. There are three validated versions of this questionnaire including the 18-item survey, which measures overall household food insecurity [27], the 10-item survey, which measures food insecurity in households without children, and the 6-item survey, which is the shortened version that only measures adult food insecurity [26]. Of the 11 studies included in this literature review, five utilized the 18-item or 6-item USHFSSM (Table 1).

Several studies included in this literature review utilized investigator-defined questions focusing on the home environment to measure food insecurity. Melchior et al. used parent reports on the following four questions: “(1) whether family members had eaten less than they should have because they had run out of food or money to buy food; (2) whether family members had eaten the same foods several times because they did not have anything else and could not afford to buy other foods; (3) whether the family could not afford to offer nutritious meals to the children; (4) how often family members did not eat as much as they should have because they had run out of food or money to buy food” [12]. Slopen et al. also utilized parental report, measuring food insecurity by relying on a positive response to, “Has there been a time when there was not enough money at home to buy food” [11]. These questions only measure one aspect of food insecurity. Therefore, these measures are not validated measures of food insecurity, but more accurately describe food insufficiency.

The Community Childhood Hunger Identification Project (CCHIP) is an assessment tool that classifies households and children as “hungry”, “at-risk for hunger”, or “not hungry” [22]. As indicated in Table 2, the CCHIP questionnaire was used to assess hunger in two studies from this literature review [22,23]. At the time of these studies, this measurement was only validated to measure hunger; however, the same questions included in the assessment were later validated to measure food insecurity [22,23,26,27].

Two studies included in this literature review developed investigator-defined questions to measure hunger by participant self-report (Table 2). Walker et al. interviewed adolescents on the frequency they experienced hunger at home during the previous year due to a lack of food [14]. Similarly, Vaughn et al. developed a single question modeled from past epidemiologic studies utilizing one-item measures for hunger by participant self-report: “How often did a parent or other adult living in your home make you go hungry or not prepare you regular meals?” [24]. However, these measures are not validated assessments of hunger.

#### 4.1.2. Psychological Assessment Methodology

The psychological assessments utilized in these studies show a substantial amount of variance in the methodology used to measure ADHD symptoms among children. The most commonly used methods are the Pediatric Symptom Checklist (PSC) and the Child Behavior Checklist (CBCL). Four studies included either the PSC or the CBCL, with one of the studies including both measures. Other studies used a variety of other methods such as the Composite International Diagnostic Interview (CIDI), the Pediatric Quality of Life Inventory (PedsQL), the Social Skills Rating Scale (SSRS), the Strength and Difficulties Questionnaire (SDQ), the Conners Teacher Rating Scale-39 (CTRS-39), the Conners Parent Rating Scale (CPRS), the Behavior and Activities Checklist (BAC), the Manifest Anxiety Questionnaire (MAQ), and the Alcohol Use Disorder and Associated Disabilities Interview Schedule-IV (AUDADIS-IV) (Table 1). A brief summary of these methods follows.

The Child Behavior Checklist (CBCL) is a questionnaire that relies on a parental report of child behavior, involving an externalizing subscale that consists of aggression and inattention/hyperactivity [30]; 3 out of 11 studies included items from the CBCL in their methodology (Table 1 and Table 2) [11,18,23]. The Pediatric Symptom Checklist (PSC) assesses child emotional and behavioral symptoms by parental report [31]; 2 out of 11 studies included the PSC in their methodology for child mental health (Table 2) [22,23]. The majority of the remaining methods used in this child literature are considered investigator-defined questions (Table 1 and Table 2) [12,13,14,20,21,24]. Only one study included in this literature relied on a diagnosis of child mental health disorder (Table 1) [19]. It is imperative to note that many of these assessment materials were not designed to specifically screen for ADHD. Thus, in order to further explore this relationship, there is a need to utilize validated and standardized measures in the assessment of pediatric ADHD symptoms.

### 4.2. Nutrition and ADHD: A Missing Link?

Over the past decade, substantial evidence has accumulated describing the neurobiological pathways involved in ADHD pathology. Dysfunctional dopamine and noradrenaline prevalence/regulation remains the primary hypothesis driving symptomology of this disorder, further validated by the effectiveness of stimulant medication, such as methylphenidate (MPH), commonly known as Ritalin, in the treatment of ADHD [32]. New evidence exists to support that MPH treatment may involve promoting alterations in amino acid pools and energy metabolism in order to normalize CNS dopamine availability, nutrient supplementation, (i.e., administration of amino acids and/or micronutrients), which serve as co-factors for the synthesis of neurotransmitters from amino acid precursors, may be an alternative method to treat ADHD. Hypothesized mechanisms of action include the role of vitamins and minerals in the methylation/methionine cycle and its subsequent involvement with neurotransmitter synthesis, DNA/RNA production, and mitigation of oxidative stress, which is frequently high in ADHD patients [33]. Further, the amino acid and neurotransmitter relationships in ADHD may hinge upon metabolomic differences or functional abnormalities, such as altered amino acid transporters or availability of multinutrients as co-factors for neurotransmitter synthesis [32].

The effects of nutrition on childhood development are becoming more widely understood, specifically in the context of brain development. Current evidence suggests that nutrition and diet may play influential roles in mental health prevalence and severity, such as with ADHD symptoms [10,20,34,35,36,37,38]. In the realm of behavioral disorders single nutrient trials have seen inconsistent results and are now less commonly explored [39,40,41]. The majority of these single nutrient trials have targeted minerals that are essential for the metabolism of neurotransmitters, such as zinc, iron, and magnesium [33,42,43].

The research to date indicates greater symptom improvement in multinurients for ADHD in comparison to single nutrient interventions. Previous adult studies have found broad-spectrum micronutrient supplement resulted in improvements in ADHD symptoms and emotional regulation after micronutrient supplementation [34,35,36,44,45]. Additional research still needs to be conducted in multinutrient interventions and ADHD to better understand these mechanisms; however, the evidence to date presents certain advantages to addressing functional nutrient deficiencies in this population with multinutrient supplementation.

The link between food insecurity and improvement in mental health symptoms likely channels through several mechanisms. Food insecure households are at an increased risk for micronutrient insufficiency or deficiency [10,46]. The experience of hunger and long-term stretches of poor food quality and quantity could contribute a critical role in this relationship [10,46]. There are several potential pathways by which food insecurity could affect ADHD symptoms. The purpose of the present literature review, however, is to summarize the current literature on food insecurity and ADHD symptoms in children thus a detailed analysis of these pathways is not included.

### 4.3. Limitations and Future Implications

Literature exploring hunger and symptoms of ADHD have found that hungry children displayed more inattentive, hyperactive, and impulsive behaviors [14,22,23,24]. This relationship is corroborated in parent and teacher reports [14,22,23,24]. Therefore, it may prove clinically useful to routinely screen children for hunger, especially for those being considered for an ADHD diagnosis, given evidence that hunger may be a key contributor to inattentive, impulsive, and hyperactive symptoms in children. This recommendation is further supported by the identification of a direct path from frequent childhood hunger to later interpersonal violence, with impulse-control deficits as a mediating factor, while controlling for sociodemographic factors such as household income, race/ethnicity, and education [24].

Limitations of this literature review are reflected in the challenges of summarizing studies utilizing various methods for measuring food insecurity, food insufficiency, hunger, and psychological assessments capable of capturing symptoms of ADHD. In addition, not all studies were able to control for potential confounders in the relationship between food insecurity and ADHD symptoms such as poverty, household income, and parental mental health [19,22,25]. Intellectual assessment questionnaires would also have been helpful for studies assessing hunger in order to guarantee this was not mediating the relationship between symptoms of inattention and hunger. Diet patterns are also widely unknown for children with ADHD, which makes extrapolations of compromised nutrition as a result of household food insecurity a challenge in determining the magnitude or causality of this relationship. In addition, the majority of these studies explore correlations between food insecurity and ADHD, but there may be other risk factors related to both food insecurity and behavioral problems in childhood that have yet to be captured in the few longitudinal analyses explained in this review. Nonetheless, this review serves as a comprehensive examination of literature on food insecurity and pediatric ADHD symptoms, highlighting the limitations of current research and discussing the need for additional investigations regarding this relationship. Future research should seek to assess this relationship using validated and appropriate measures in order to help establish the magnitude of the effect of food insecurity on ADHD symptoms.

## 5. Conclusions

In summary, the literature to date provides substantial evidence for an inverse relationship between the presence of childhood food insecurity and ADHD symptoms. Results indicate food insecurity and other challenges related to adequate food intake impacts pediatric ADHD symptoms with possible lasting effects into adulthood. Future research, with validated and consistent assessment methods, is needed to examine the consequential impacts of food insecurity on early behavioral development.

## Figures and Tables

**Table 1:** Comparison of literature characterizing associations between food insecurity and symptoms of Attention-Deficit Hyperactivity Disorder (ADHD) in a pediatric population.

Author, Year	Population	Assessment Methods		Results
		Food Insecurity	Psychological	
Whitaker, 2006 [18]	US children ages 0–3 years (*n* = 2870)	USHFSSM ^1^ Parent Report	Parent report based on CBCL ^2^ with categories for hyperactivity/inattention.	Behavior problems among children of fully food insecure mothers remained significantly elevated, with the percentage of children with behavior problems increasing with worsening household food insecurity.
McLaughlin, 2012 [19]	US adolescents ages 13–17 years (*n* = 6483)	6-item USHFSSM ^1^ Parent Report	Modified CIDI ^3^ Adolescent Interview resulting in ADHD Diagnosis	Higher food insecurity scores were associated with 14% greater odds of past-year mental disorders, including ADHD, in adolescents.
Kirk, 2015 [20]	Canadian children ages 10–11 years (*n* = 5853)	6-item USHFSSM ^1^ Parent Report	Child questionnaire using investigator-defined questions based on PedsQL ^4^	Marginally, moderately, and severely food insecure children were more likely to report problems with mood and externalizing problems such as inattention.
Kimbro, 2015 [13]	US Children grade K-1 (*n* = 6300)	18-item USHFSSM ^1^ Parent Report	Teacher report using SSRS ^5^	Teachers reported poorer scores for externalizing behaviors and self-control in children transitioning into food insecurity.
Poole-Di Salvo, 2016 [21]	US adolescents ages 12–16 years (*n* = 8600)	18-item USHFSSM ^1^ Parent Report	Parent report using SDQ ^6^ with categories for hyperactivity and conduct problems^+^	Parent-reported scores for hyperactivity were significantly higher in adolescents in food insecure households.
Slopen, 2010 [11]	US children ages 4–14 years (*n* = 2810)	Parent Report: positive response to question “has there been a time when there was not enough money at home to buy food.”	Parent Report: CBCL ^2^ including aggression, hyperactivity, and non-compliance	Symptoms of hyperactivity are more prevalent in children living in food insecure households, with data suggesting that transitions into food insecurity are more likely to predict behavioral problems than chronic food insecurity.
Melchior, 2012 [12]	Canadian children ages 1.5–8 years (*n* = 2120)	Parent Report: 4 questions regarding food insecurity asked at 1.5 years and 4.5 years	Parent Report: A combination of psychological questionnaires and investigator-defined questions on aggression, hyperactivity and aggression	Food insecurity distinctively predicted children’s two-fold increased likelihood of persistent hyperactivity and inattention, even after controlling child and family characteristics.

^1^ USHFSSM = United States Household Food Security Survey Module; ^2^ CBCL = Child Behavior Checklist; ^3^ CIDI = Composite International Diagnostic Interview; ^4^ PedsQL = Pediatric Quality of Life Inventory^™^; ^5^ SSRS = Social Skills Rating System; ^6^ SDQ = Strengths and Difficulties Questionnaire.

**Table 2:** Comparison of literature characterizing associations between hunger and symptoms of ADHD in a pediatric population.

Author, Year	Population	Assessment Methods		Results
		Hunger	Psychological	
Kleinman, 1998 [22]	US children ages 6–12 years (*n* = 328)	Parent Report: 8-item adult CCHIP ^1^ hunger scale	Parent Report: PSC ^2^	Hungry children scored higher in inattention and hyperactive/impulsive symptoms and were 3-times more likely to be classified as dysfunctional (scoring 28 or higher on PSC) by parent report.
Murphy, 1998 [23]	US children ages 6–12 years (*n* = 205)	Parent Report: 8-item adult CCHIP ^1^ hunger scale; Child Report: 5-item child CCHIP ^1^ hunger scale	Parent Report: PSC ^2^, CBCL Teacher Report: CTRS-39 ^3^	Teachers reported hungry and at-risk of being hungry children more likely of having higher levels of hyperactivity and attention problems.
Walker, 2007 [14]	Jamaican adolescents ages 17–18 years (*n* = 129)	Adolescent Report: interviewed on frequency of hunger experienced in the home during the previous year due to lack of food	Parent Report: short form CPRS ^4^Adolescent Report: Antisocial Behavior: BAC ^5^ Anxiety Symptoms: MAQ ^6^	Hungry adolescents had poorer psychological functioning in late adolescence, with parents reporting more hyperactivity.
Vaughn, 2016 [24]	US young adults 18+ years (*n* = 34,427)	Participant Report: fairly often or often response to “how often did a parent or other adult living in your home make you go hungry or not prepare you regular meals”	Participant Report: Psychiatric Disorders: interviewed with AUDADIS-IV ^7^	Participants who experienced childhood hunger were more likely to report challenges related to impulse control, with impulse control deficits mediating the relationship between frequent childhood hunger and later interpersonal violence.

^1^ CCHIP = Community Childhood Hunger Identification Project; ^2^ PSC = Pediatric Symptom Checklist; ^3^ CTRS-39 = Conners Teacher Rating Scale-39; ^4^ CPRS = Conners Parent Rating Scale; ^5^ BAC = Behavior and Activities Checklist; ^6^ MAQ = Manifest Anxiety Questionnaire; ^7^ AUDADIS-IV = Alcohol Use Disorder and Associated Disabilities Interview Schedule-IV.
